# An Analytics Environment Architecture for Industrial Cyber-Physical Systems Big Data Solutions

**DOI:** 10.3390/s21134282

**Published:** 2021-06-23

**Authors:** Eduardo A. Hinojosa-Palafox, Oscar M. Rodríguez-Elías, José A. Hoyo-Montaño, Jesús H. Pacheco-Ramírez, José M. Nieto-Jalil

**Affiliations:** 1 División de Estudios de Posgrado e Investigación, Tecnológico Nacional de México/I. T. de Hermosillo, Hermosillo, Sonora 83170, Mexico; d02330027@hermosillo.tecnm.mx (E.A.H.-P.); jose.hoyom@hermosillo.tecnm.mx (J.A.H.-M.); 2 Departamento de Ingeniería Industrial, Universidad de Sonora, Hermosillo, Sonora 83000, Mexico; jesus.pacheco@unison.mx; 3 Escuela de Ingeniería y Ciencias, ITESM Sonora, Hermosillo, Sonora 83000, Mexico; jnietoj@tec.mx

**Keywords:** analytics environment architecture, industrial Big Data analytics, industrial Big Data architecture design, CPS analytics

## Abstract

The architecture design of industrial data analytics system addresses industrial process challenges and the design phase of the industrial Big Data management drivers that consider the novel paradigm in integrating Big Data technologies into industrial cyber-physical systems (iCPS). The goal of this paper is to support the design of analytics Big Data solutions for iCPS for the modeling of data elements, predictive analysis, inference of the key performance indicators, and real-time analytics, through the proposal of an architecture that will support the integration from IIoT environment, communications, and the cloud in the iCPS. An attribute driven design (ADD) approach has been adopted for architectural design gathering requirements from smart production planning, manufacturing process monitoring, and active preventive maintenance, repair, and overhaul (MRO) scenarios. Data management drivers presented consider new Big Data modeling analytics techniques that show data is an invaluable asset in iCPS. An architectural design reference for a Big Data analytics architecture is proposed. The before-mentioned architecture supports the Industrial Internet of Things (IIoT) environment, communications, and the cloud in the iCPS context. A fault diagnosis case study illustrates how the reference architecture is applied to meet the functional and quality requirements for Big Data analytics in iCPS.

## 1. Introduction

The Internet of Things (IoT) has been widely adopted by the industry [1], and its impact is transforming manufacturing. The Industrial Internet of Things (IIoT) consists of creating networks of physical objects, environments, vehicles, and machines through integrated electronic devices that allow the collection and exchange of data [2]. It opens the possibility of creation of industrial cyber-physical systems (iCPS) with the convergence of information technology about networks, connectivity, data, ecosystems, and information systems with operational technology talking about physical plant equipment, machinery, and systems for monitoring and controlling. The construction of iCPS enables the integration of information with manufacturing processes [3]. In this context, iCPS connect machines, systems, and assets, so organizations can create smart networks along the value chain to, among other things, optimize production processes [4]. All this interconnection of objects that generate and process data and information also require new approaches to better manage this exponential growth of data and information to take advantage of this exponential data accumulation [5].

Machine learning models are used in Big Data to identify complex patterns to extract information from large amounts of data from industrial processes under observation [6]. Even though sophisticated data analysis is required, a data model design approach to allow the development of analytics systems architectures for the industry is necessary [7].

Different architectural approaches can be found in architectural design to address specific technologies or particular areas, but there is a lack of Big Data applications that integrates an analytics environment for iCPS. However, industrial analytics receives the most research interest [8,9,10].

Big Data solutions for iCPS need to integrate technologies in a consistent ecosystem in an industrial environment, but such solutions are complex. A reference architecture guide for Big Data analytics could facilitate the development, implementation, and operation of Big Data iCPS solutions in the industry [11]. This paper extends and completes a preliminary proposal to address this problem [12], particularly the design of a reference architecture in industry 4.0 that follows better architectural design practices of software engineering to facilitate the design and implementation of software-based solutions with a focus on data management.

This paper aims at presenting an improvement in the design of Big Data analytics solutions for iCPS, and to provide support for the modeling of data elements, predictive analysis, inference of the key performance indicators, and real-time analytics, through the proposal of an architecture that will support the integration from IIoT environment, communications, and the cloud in the iCPS. It also presents the design of software-based solutions for iCPS with a case study for the fault diagnosis system domain that illustrates Big Data analytics on industrial cyber-physical systems.

The remainder of this paper is structured as follows: Section 2 presents the state of the art of Big Data analytics in iCPS. Section 3 deals with general concepts related to industrial Big Data. Section 4 presents a design framework for data management architecture, which precedes a fault diagnosis scenario for optimization data services, real-time equipment monitoring, and forecasting unusual process conditions in Section 5. A discussion of the main findings together with their limitations and future work is presented in Section 6. Finally, the conclusion of this paper is in Section 7. For future reference, a list of acronyms used in this paper is provided in Table 1.

## 2. Related Works

The architectural design of Big Data solutions follows the challenges from IIoT demanding ecosystem with cloud services. Additionally, including the challenges to provide context to support data analysis. Such requirements require an agile and flexible architecture integration; hence, a reference architecture proposal is a common approach of various researcher’s architecture design. For example, in [13], the architectural design focus is emphasized, pointing out the complexity in Big Data management versus small data system differences. Big Data-system design (BDD) addresses the design of Big Data architecture combining data modeling with reference architectures and technology catalog to facilitate the development of a fitting Big Data platform development. In [7], a solution for analysis of goal and obstacle for Big Data architecture is proposed, selecting architectural decisions using imperfect information that satisfies goal drivers of stakeholders at the moment of the decision outcome. In [14], a use case modeling method to make an abstraction of the Big Data architecture to describe the role, activities, and functional components of Big Data in a better and more neutral way is presented.

The development of architectures of Big Data analytics that consider the different points of view in the technological merge that occurs in iCPS and allows the development of Big Data analytics platforms is another of the approaches of several researchers. For example, an architecture that collects and integrates industrial data from IIoT and manufacturing information systems (MIS) is proposed in [15] with an approach for the model-driven generation of data integration architectures to enable subsequent industrial analytics. A five-level architecture is used for CPS design in [10,16]. The architecture is called 5 C because it is built in five levels, which are intelligent connection, data to information conversion, cybernetic, cognition, and configuration. This five-level architecture is presented as an aid to guide the development of iCPS, aiming for smart manufacturing and resilient equipment that proves quality products and enhanced system performance.

An architecture for the entire life cycle of aluminum industry production and supply chain in Industry 4.0 is presented in [17], it is a theoretical approach of an architecture composed of nine layers: seven horizontal layers (from physical equipment to data analysis) and two vertical layers (data models and software systems), each one containing several components and subcomponents, making the architecture too complex, since it is focused on integrating all the physical and software elements and systems that could be in operation in an aluminum production company. In [18], the cognitive oriented IoT Big-data (COIB) Framework integrates Big Data and IoT to implement an industrial IoT system. The COIB-framework wrangles data to extract the right data and metadata from multisource and heterogeneous data for better data governance. In [19], the authors propound a data architecture based on five layers that attempt to integrate sensors, actuators, networks, the cloud, and technologies of IoT for the generation of applications for Industry 4.0. The data response layer maintains persistence among the others through data management between them.

The evolution of manufacturing execution systems (MES) from traditional manufacturing to industry 4.0 integrates manufacturing activities with information technology advancements that rely on real-time software applications. For example, an MES system that connects the world machines and business systems that consider the particular techniques of production systems are proposed in [20] with the personalization of software for such automation of production systems. An approach for automation on the integration of MES with other manufacturing systems that simplify the design and implementation in the food and beverage industry is proposed in [21]. The approach in [22] focuses on real-time analytics for activities on the shop floor. Human tasks can be automated or augmented by the new role of MES in the digital twin of a physical product.

The analysis of the selected articles indicates a lack of development of applications of industrial analytics. Therefore, given the need for analytics in the industry is growing fast and considering that industrial Big Data is still in an early stage, this opens the possibility of a contribution that considers an architecture design that integrates the Big Data technologies with industrial analytics.

## 3. Industrial Big Data Analytics

**Figure 1:**
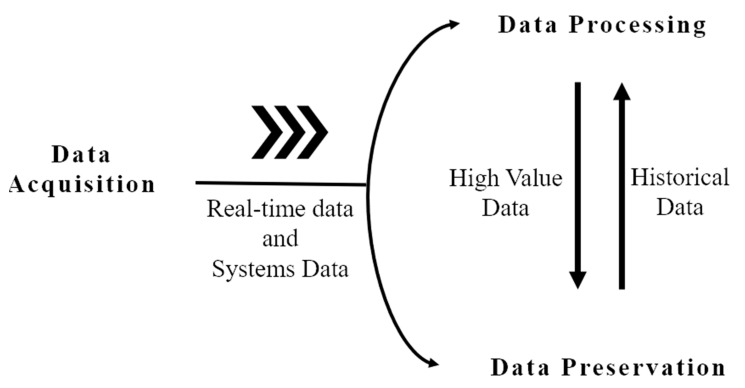
Big Data lifecycle.

In iCPS, data is generated by multiple sources in different contexts such as facilities equipment, process equipment, manufacturing systems, and environment data, including sensors, machine controllers, manufacturing systems, just to mention a few. This could generate a massive volume of data that arrives at high speed and with different formats, called Big Data (see Figure 1).

However, raw data is useless to get the data value. Firstly, data wrangling for iCPS is needed before processing because of data noise, multiformat, scale differences, heterogeneous sources, among other issues. Next, high-value data is preserved as historical data and processed for exchanging and sharing at all levels. Then, usually through cloud services, data are processed by data mining and machine learning.

**Figure 2:**
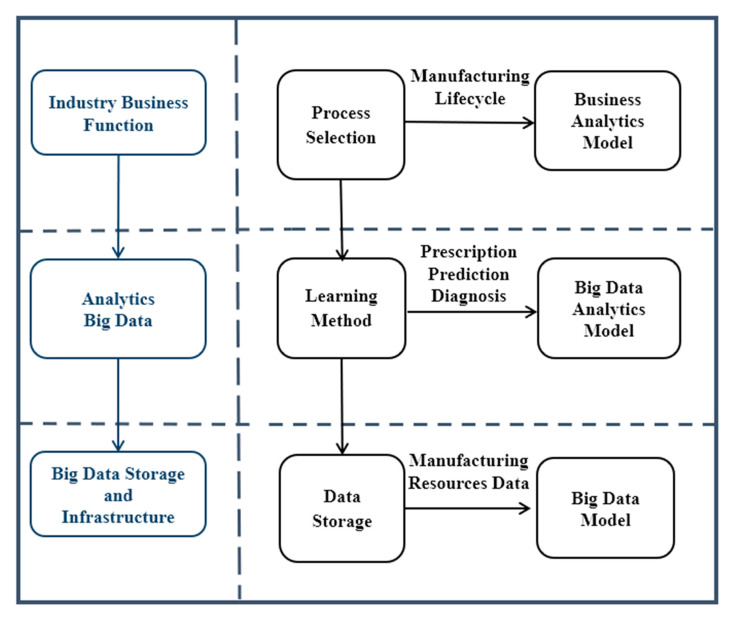
Big Data analytics management model.

To address the last challenges, an analytics architecture for iCPS Big Data that considers the novel paradigm in the integration of Big Data technologies into iCPS and allows the creation of applications from data models (see Figure 2), that support Big Data analytics and address industrial challenges, even though Big Data technology rapidly changes, were defined following three questions. The first question is how to address industrial process challenges to achieve a Big Data model, and how Big Data systems carry out the design phase of the industrial Big Data management drivers? The architecture design of industrial analytics system is much more critical than it is for legacy data systems, so the second question centers on how to extend traditional design architecture for the system design of iCPS analytics? A third question is the integration of the other two: how can the Big Data model and architecture design be integrated for analytics system framework in the context of iCPS?

An architectonical guide should consider new data modeling analytics techniques that include:

- Diagnosis modeling. It extracts information from historical and transactional industrial data processes to recognize patterns in data by employing pattern recognition approaches.
- Predictive modeling. It uses Big Data mining by applying modern techniques of statistical approaches, data visualization, and pattern recognition approach to recognize organization threats and forecasts.
- Prescriptive modeling. It is used to generate better solutions by means of real-time operation machine learning-based optimization models that allow data integration and processing, enabling real-time monitoring and to make better decisions.

### 3.1. Industrial Big Data Management Drivers

Data is an invaluable asset in iCPS [23]. It enables smart manufacturing. Its strategic significance is to obtain the value for analytics through Big Data processing.

Before the production process begins, the smart production plans conduct the consideration of resource data of the production process. Next, the relationship of global data allows the optimized production plan to be quickly generated, improving planning speed and accuracy.

In the production process, real-time data should facilitate the monitoring of the production process, so the manufacturers could keep up to date with the production deviations to generate optimal operational control plans [24]. Fault diagnosis and operation process optimization can be accomplished by storing and analyzing data for active preventive maintenance, repair, and overhaul (MRO), through Big Data from IIoT.

**Figure 3:**
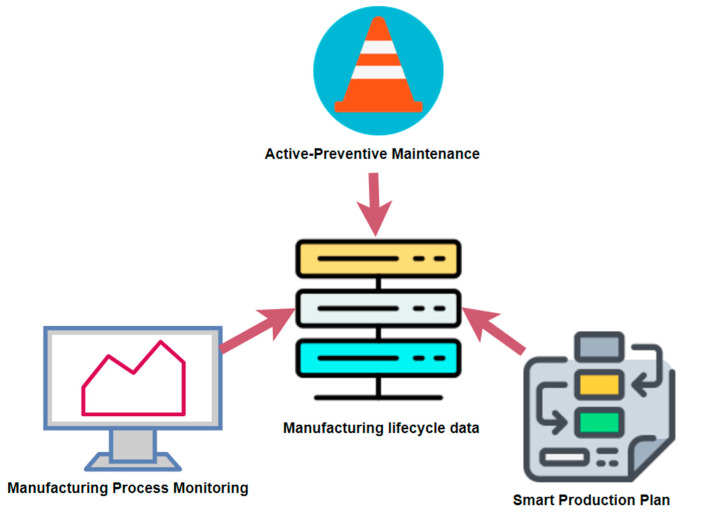
Manufacturing lifecycle data. (Graphics source: https://online.visual-paradigm.com/, accessed date: 14 June 2021).

Figure 3 shows that in iCPS, Big Data emerges from the accumulation of data generated in the manufacturing lifecycle, such as planning, production, and MRO [25].

A simplified data source for industrial analytics for iCPS useful for understanding industrial Big Data management drivers is shown below, including real-time data for industrial processes and data for manufacturing information systems. In [23] a complete classification of data types for smart manufacturing can be found.

- Resource data from production processes, which includes (a) data collected from IIoT infrastructure; (b) data collected from material, product, and service systems; (c) environmental data.
- Management data from manufacturing information systems (manufacturing execution system (MES), enterprise resource planning (ERP), customer relationship management (CRM), supply chain management (SCM), precedence diagram method (PDM)), computer-aided systems (CAS), computer-aided design (CAD), computer-aided engineering (CAE), and computer-aided manufacturing (CAM)).

### 3.2. Industrial Big Data Attributes

**Figure 4:**
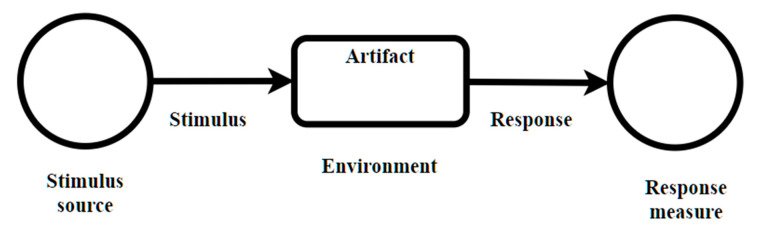
Scenario definition.

The scenario technique focuses on identifying stimulus and how the system should respond to it. It is also related to quality attributes and seeks to highlight the consequences of architectural decisions encapsulated in the design. Quality attributes in this context are related to the design of software products, where these attributes are related to functional requirements that the architecture must satisfy. However, data quality attributes, although an important issue in manufacturing quality, is out of the scope of this work, and they are covered in other works such as [26]. The scheme proposed by [27], presented in Figure 4, describes a quality attribute. The stimulus describes an event that reaches the system and represents a condition that requires a response. The stimulus source can affect the way the stimulus is treated by the system. The response is the activity carried out in response to the arrival of a stimulus. The measurement of the response allows determining if the requirement was satisfied. The artifact is the portion of the system that applies the requirement. The environment is the set of circumstances in which the stimulus is carried out.

**Figure 5:**
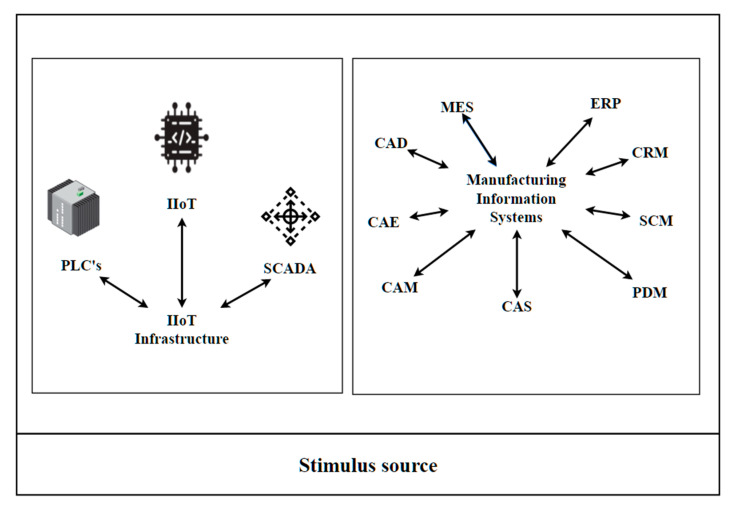
Stimulus source for the main attribute scenarios. (Graphics source: https://online.visual-paradigm.com/, accessed date: 14 June 2021).

**Figure 6:**
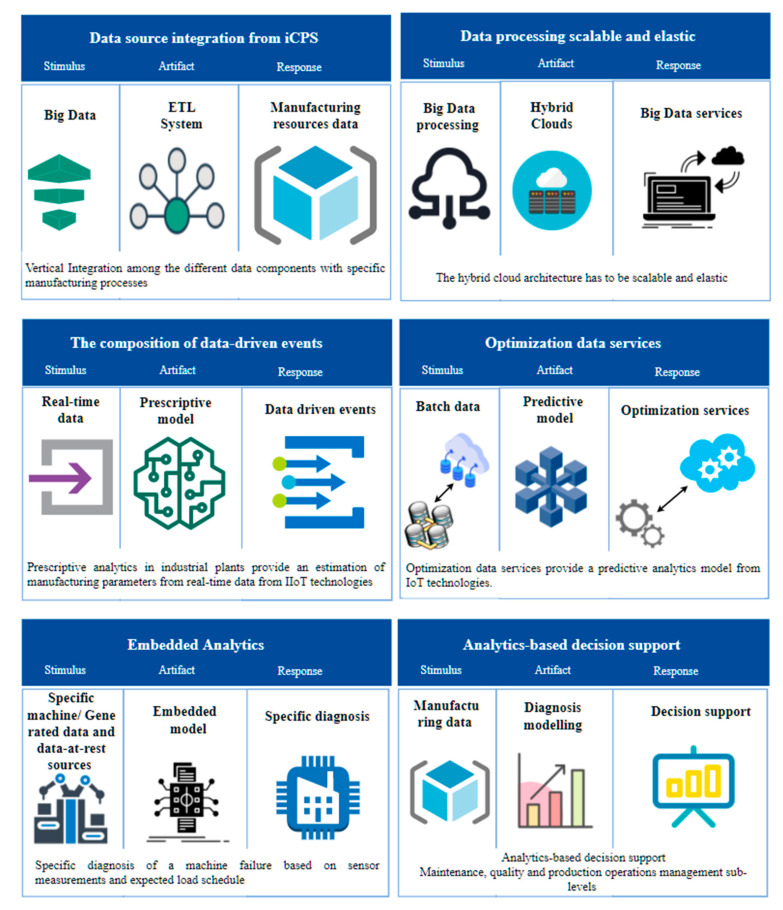
Scenario definition. (Graphics source: https://online.visual-paradigm.com/, accessed date: 14 June 2021).

Six main scenarios were considered to identify quality attributes to illustrate the characteristics of interest for data management in Industry 4.0. Those scenarios were first proposed in [12] and were defined following the scenario technique for quality architectural requirements identification and description [28]. Figure 5 describes the stimulus source based on manufacturing lifecycle data that is the same for the six scenarios. Figure 6 shows the scenario definition for each industrial Big Data attribute. Based on the above, the quality attributes are defined in Table 2.

## 4. Industrial Big Data Design Architecture for Analytics

Different methodologies propose distinct approaches for the development of software architectures [29], most of them covering the architecture lifecycle defining the structure of the system at a high level but present few specifications on how to do the design activity. The attribute-driven design approach (ADD) is the first software methodology based on the design of quality attributes to satisfy different scenarios by selecting architectural structures and their representation into views. ADD takes the requirements, quality, and functionality as input and as output produces a conceptual architecture that provides the basis for the architecture design. It also includes architecture analysis and documentation as an integral part of the design process [30]. A detailed architecture may require refining sketches created during early design iterations and then joining those design decisions to implementation options accessible through frameworks. Additionally, ADD extends to use reference architectures matching with a catalog of technology ratings including tactics, patterns, and frameworks.

**Figure 7:**
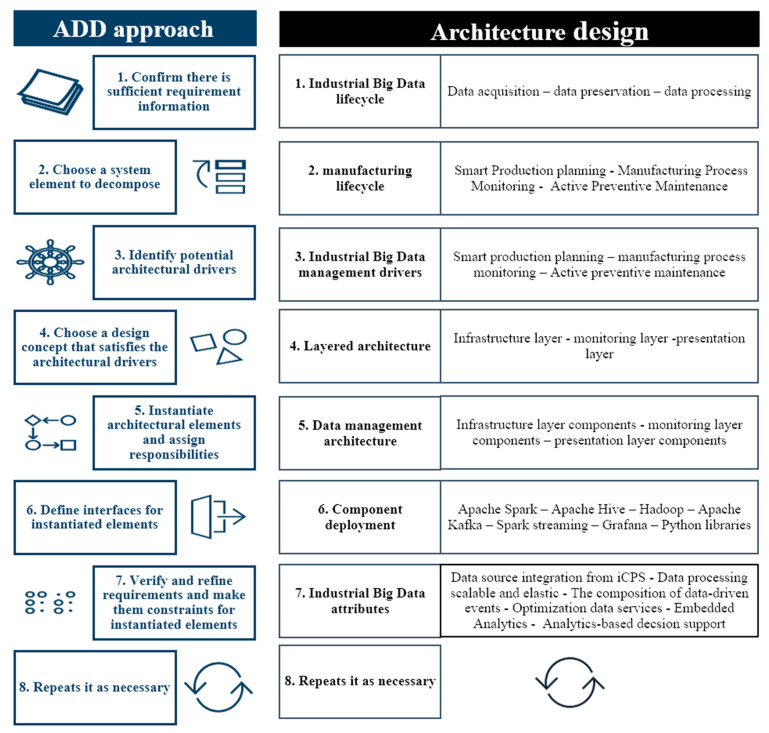
The process of attribute-driven design approach. (Graphics source: image library of Microsoft Office).

The ADD process consists of seven stages (see Figure 7), beginning with input identifications for the design of the architecture. These inputs are the design purpose, the main functional requirements, the main scenarios of quality attributes, constraints, and architectural concerns. With the review of mentioned entries, the first step is to confirm there is sufficient requirement information through the industrial Big Data lifecycle: data acquisition–data preservation–data processing.

The second step is to choose a system element to split through the manufacturing lifecycle (smart production planning–manufacturing process monitoring–active preventive maintenance).

In the third step, potential industrial Big Data management drivers are identified, ranking the requirements based on their impact on the architecture (industry business function, analytics Big Data, Big Data storage, and infrastructure).

Step four involves choosing a design concept that satisfies the architectural drivers. A layered architecture is adopted to fulfill the requirements of industrial Big Data analytics attributes (infrastructure layer, monitoring layer, presentation layer).

In step five, elements of the architecture are instantiated, and responsibilities are assigned (infrastructure layer components, monitoring layer components, presentation layer components).

In step six, interfaces are defined for instantiated elements through the component deployment (Apache Spark, Apache Hive, Hadoop, Apache Kafka, Spark streaming, Grafana, and Python libraries).

Step seven includes verifying and refining requirements and making them constraints for architecture elements instantiated (data source integration from iCPS, data processing scalable and elastic, composition of data-driven events, optimization data services, embedded analytics, analytics based decision support). Finally, it is repeated as required.

### 4.1. Data Management Architecture

**Figure 8:**
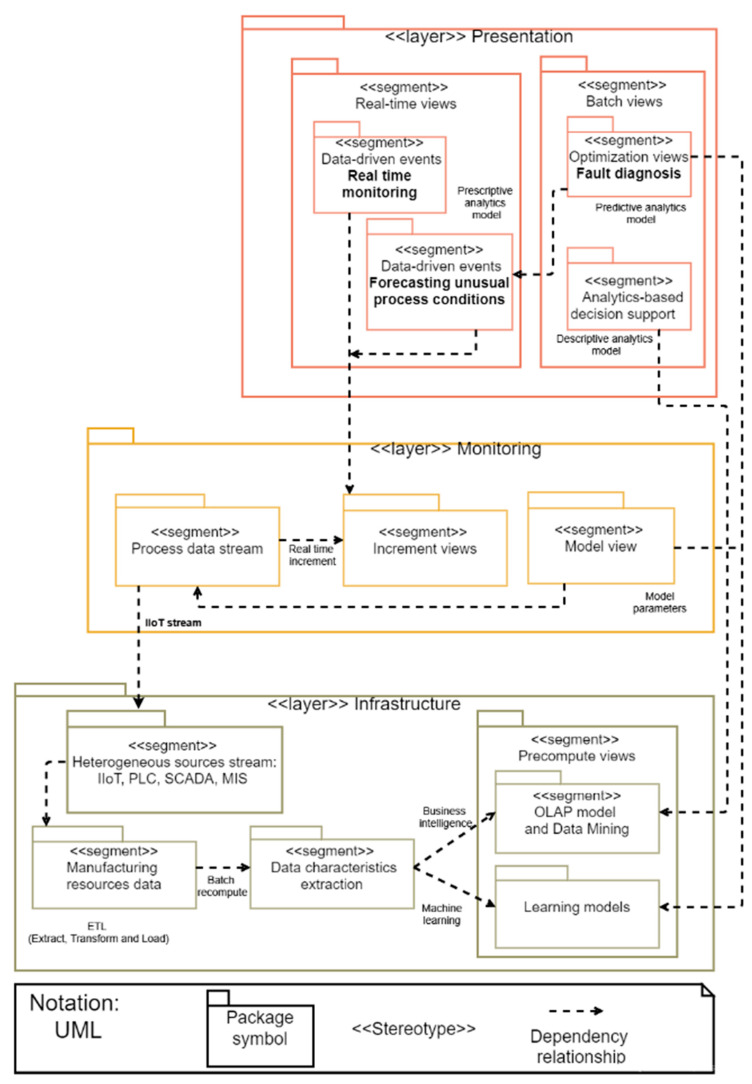
Layered data management architecture for iCPS Big Data analytics in Industry 4.0.

A layered architecture is adopted to fulfill the requirements of industrial Big Data analytics attributes, and to be able to handle a wide range of workloads and industrial scenarios, where low read and write latency are required [12]. This Big Data architecture is composed of three layers of components that attempt to solve the problem of calculating functions in real-time, as depicted in Figure 8.

1. An infrastructure layer that manages an immutable master data set and only calculates query functions called views lots,
2. A monitoring layer that compensates for the high latency of data actualization to the presentation layer and only processes recent data, and
3. A presentation layer that indexes batch views for low-latency ad hoc queries.

### 4.2. Infrastructure Layer

The manufacturing resource data component stores Big Data from low data ingestion (for example, data from transactional databases), or data with high data ingestion (like, for example, data streams from sensors), like a historic, append-only set of raw data. Then a list of features suitable for applying the respective model for the learning algorithms is obtained by processing data. If there are criteria changes, data are reprocessed.

In the learning model component, the parameters describing the data model depend on the machine learning methods used. Anomaly detection would include time stamp aggregates of manufacturing data operation. In the context of processing large data sets, it is significant to use distributed data processing depending on a large degree of the actual data and learning method used.

The OLAP model and Big Data mining component through Big Data warehouse storage allow the multidimensional data model (MDM) that enables data analysis of large volumes of structured data to support decision-making processes in a business intelligence context.

### 4.3. Monitoring Layer

The monitoring layer ingests a new data stream from IIoT. Its main function is monitoring the incoming data in current time. It deals with data processing in real-time that is usually time-dependent, so it is crucial to reduce latencies added by accessing the saved learning model in the presentation layer. The process stream component processes time-series data to obtain the features out of the new data stream. In the increment view component, the continuous measurement over time represents an important function in identifying data outliers, referring to the fact that the patterns are not supposed to change abruptly except unusual work data occurring in processes, then transferring the obtained model input to the real-time monitoring component in the presentation layer in the matching time period, with the result of the learning model and if the threshold is exceeded, and is detected consecutively over some time a data event is presenting in the real-time view. Even though sensor errors or other data imprecisions may occur is not required to disseminate an anomaly event. Therefore, the anomaly event takes place if outliers are determined sequentially over a specific time.

### 4.4. Presentation Layer

The presentation layer shows a view of the output of data patterns produced by the layer functions of infrastructure and presentation through real-time and batch views. Thus, the real-time monitoring component analyzes the delivery of continuously updated information to identify anomalies in aggregated data over time from manufacturing operations from the increment views component, to identify serious problems using the learning model parameters from the fault detection model estimated in the model view component. Furthermore, forecasting unusual process conditions component predicts problems of process performance analyzing data, tracing recurrent patterns, and optimization views from the predictive analytics model are used to flag abnormal behaviors, applying data-driven events to proactive monitoring. In the batch views, the fault diagnosis component presents predictive analysis to improve anomaly forecasting and diagnosis of manufacturing systems, with optimization views of process equipment and facilities equipment based on Big Data mining methods implemented in the learning models component. The analytics-based decision support component uses batch views to supports on-line analytical processing (OLAP) analysis to inform, observe, and show how big or small the problem is, using a Big Data warehouse from the infrastructure layer.

The figure also shows how the characteristics required in Industry 4.0 are related to the different components of the architecture. Multisource integration is done with coordination with the architectural components of the process data stream, heterogeneous stream, and manufacturing resources data. Scalable and elastic data processing occurs in the component of manufacturing resources data. The composition of data-driven events is done in the real-time monitoring, increment views and model view. Optimization data services are performed by the incremental view component and model views component. Embedded analytics and analysis-based decision support are calculated in precompute views and presented in the batch view component.

### 4.5. Component Deployment

**Figure 9:**
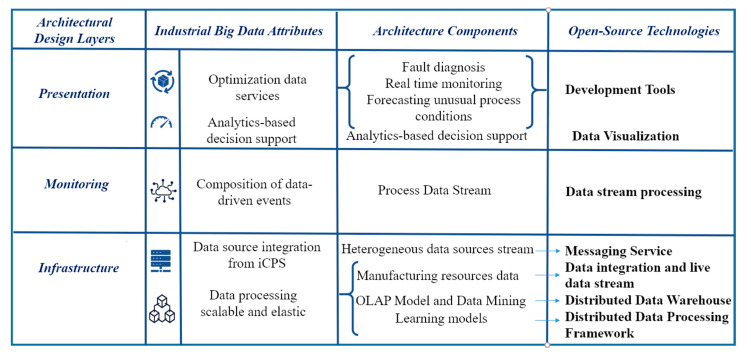
Deployment of component technologies. (Graphics source: image library of Microsoft Office).

Nowadays, different technologies for Big Data analytics are being developed, often overlapping functional requirements and quality data management attributes. For Big Data architects of iCPS systems, the selection of technology is a challenging question that requires the attention of many implementation details and compatibility constraints. Figure 9 shows technologies that are accessible under an open-source license.

Once data is available, the data characteristics extraction component is used for precomputing views of machine learning or business intelligence purposes. A distributed data warehouse through Hive enables the OLAP and data mining component.

However, Hive was developed on HDFS to enable distributed storage and processing for collecting large data sets. The learning model component uses data stored in Hadoop to build learning models using the distributed Data processing framework Apache Spark, an analytics engine for Big Data that enables better performance using the read access memory (RAM) of the server to process machine-learning algorithms.

For data monitoring purposes, the process data stream component obtains IIoT data available with Spark Streaming interacting with Apache Kafka, offering scalable, high-throughput, fault-tolerant stream processing of live data stream processing for the composition of data-driven events. Then the data is stored or pushed to the data-driven event component for real-time analysis.

For data visualization, after processing data for analytics use with development tools, Grafana integrates the visualized data for presentation purposes. The component for real-time monitoring uses the Spark Streaming processing of data-driven events to prepare data for Grafana. Additionally, the decision support component takes OLAP data from Hive and outputs over Grafana. Finally, in the same way, the fault diagnosis component uses development tools based on the python libraries to process machine learning algorithms over Big Data stored in Hadoop to prepare data for Grafana.

Figure 9 also shows the architecture components that satisfy industrial Big Data attributes. They are the characteristics required in the analytics system for Industry 4.0.

- The multisource integration requirement is fulfilled in the infrastructure layer with the following components: the architectural components of the heterogeneous stream, process data stream, and manufacturing resources data.
- Scalable and elastic data processing requirement is fulfilled in the infrastructure layer with the manufacturing resources data component.
- The composition of data-driven events is fulfilled in the monitoring layer with the following components: real-time monitoring, increment views, and model view.
- Optimization data services are fulfilled by the incremental view component and model views component.
- Embedded analytics and analysis-based decision support are fulfilled in precompute views and the component of batch view.

## 5. Case Study: Fault Diagnosis

A notably stagnant trend in the reviewed literature is the lack of applications of iCPS analytics. The data models, learning methods, and architectures are somewhat different from other areas relating to maintenance and diagnosis. Implementing Big Data Analytics architecture in factories offers several advantages. Consider a factory that searches providing self-awareness and self-prediction of machine data, controlling parameters to monitor the status, and providing the self-comparison capability into a production line consisting of numerous amounts of machine tools. This level of knowledge guarantees near-zero downtime production.

The fault diagnosis scenarios are selected to illustrate how Big Data analytics architecture is applied to meet the functional and quality requirements designing an architecture analytics solution in iCPS that fulfills the industrial Big Data attributes presented in Table 3. Scenario-based architectural evaluation methods are a well-established approach to validate the architectural design and to analyze the decisions that have been made to achieve the design approach [31]. They are thorough and comprehensive approaches that gather the stakeholders of a system and guide them through a structured process that explores the architectural design options and the resulting implications.

**Figure 10:**
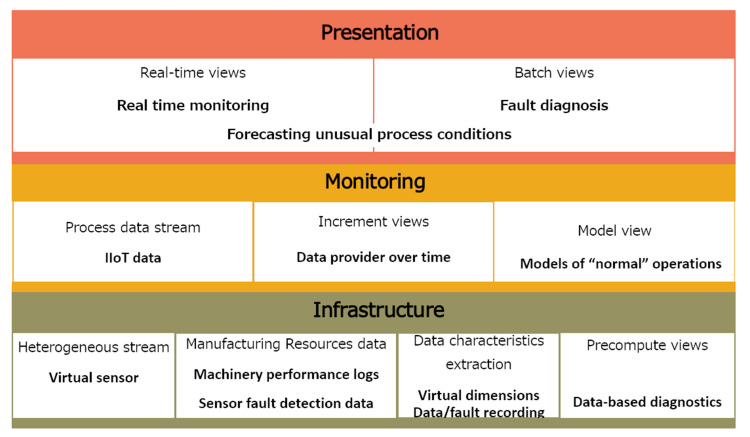
Architectural instance for industrial-analytics Big Data for the use case of fault diagnosis.

The purpose of the scenarios is to show architectural decision sensitivity and tradeoff points of architectural design for fault diagnosis in industrial plants through an architectural instance for industrial-analytics Big Data for the use case of fault diagnosis presented in Figure 10.

To better illustrate the real-world applicability of the proposed architecture, a set of real cases extracted from research literature were chosen as a means to show how the components of the architecture fit with the main elements proposed to provide solutions to real situations. The scenarios provide a view of the possible fitting of the solutions proposed to the components of the architecture, hypothetically considering the solutions as if they were implemented following the proposed architecture. The works selected are related to the next three application scenarios:

1. Fault diagnosis—shows the benefits and fulfills the data quality attribute named optimization data services.
2. Real-time monitoring in iCPS—shows the benefits and fulfills the data quality attribute named the composition of data-driven events.
3. Forecasting unusual process conditions—shows the benefits of data quality attributes named optimization data services and data-driven events.

**Figure 11:**
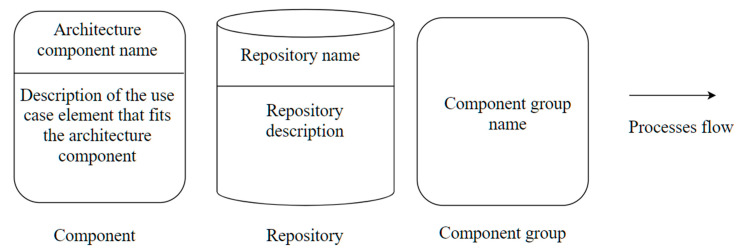
Notation icons for the elements of the process view diagram.

Figure 11 shows the notation proposed to represent architecture components, component groups, repositories, and process flows. These notation icons are used in the different diagrams of process view to describe interactions between the architecture components and the elements that fit the architecture components with the use case elements presented below. The component icon describes elements that fit an architecture component for the use case presented. The components group icon represents several components related to each other. The repository icon represents the area of data, models, or views. Finally, the process flow icon describes the sequence of the process view diagram.

### 5.1. Fault Diagnosis

Fault diagnosis plays a critical role in industrial systems. A Big Data industrial application for fault diagnosis helps increase process efficiency, prevent accidents, and saves costs. Unfortunately, in a traditional plant, to have a total understanding of the system to be optimized is almost impossible because such systems are composed of numerous components and operate in a variety of conditions. However, most of the available data contain records of incomplete or missing faults due to human factors or monitoring systems that provide inconsistent data (e.g., obsolete format).

In the optimization data services requirement, the development of a high-performance fault diagnosis system for iCPS requires mainly two types of information: first, a thorough understanding of the target system, and second, condition monitoring data/fault recording. A broad level of knowledge about system failures (i.e., mechanisms, fundamental causes) can facilitate the effective failure diagnosis for industrial plant systems.

On the other hand, the industry has many machine tools, this way time-machine records from sensors’ data of critical components can be used for prescriptive analytics models to provide self-diagnosis. Therefore, a significant amount of fault monitoring through log data, if available, can provide excellent information for data-based diagnostics.

A data-based diagnostic is a common approach that can be applied in different industrial contexts for fault diagnosis. This can be illustrated with the following examples in real scenarios: in [32] an intelligent fault diagnosis approach for a mechanical problem using Big Data and unsupervised feature learning for providing an accurate prediction in the case of motor bearings logs is presented. An additional data-based fault diagnosis example related to the petroleum industry is presented in [33], where it was observed that regular maintenance cannot effectively detect faults in reciprocating compressors. In such work, machine learning methods were applied to analyze data and to diagnose faults to predict potential faults in compressors.

To illustrate one of many possible applications of the proposed architecture for Big Data analytics in the industry for fault diagnosis scenarios, a real case for the automation problem of the machining process is discussed below, where an effective monitoring system of the condition of the face milling tool is required. In [34] a machine learning approach is presented for faulty conditions based on cutting process data to control the machining process, feeding optimized data to the machine controller. The system aims for a long machine tool life and processing quality to ensure high productivity.

**Figure 12:**
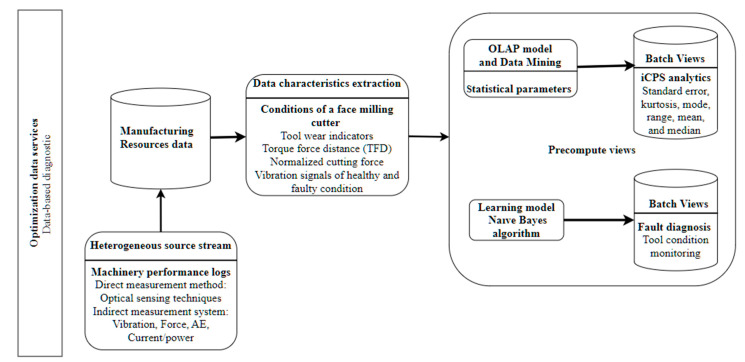
Instance of the fault diagnosis process.

The fitting of the proposed architecture to the machining process with Big Data support and machine learning is described following the process view of the architecture proposed in Section 4.1. The process view describes interactions between the architecture components for industrial analytics and the activities that realize managing Big Data during its life cycle. Figure 12 shows the instances of the architecture components that would interact during a fault diagnosis process scenario. From the analysis of the scenario used in [34], the mapping of the solution proposed to the components of the architecture is as follows: at architecture component *manufacturing resources data*, historical data from the component *heterogeneous source stream* is stored. That stored data includes machinery performance logs from the measurement method. The information extraction process is performed at the component *data characteristics extraction* by virtual dimensions that capture the conditions of a face milling cutter and monitoring data-fault recording. The *precompute views* component performs the extraction of information through diagnosis modeling or using predictive modeling. The component *OLAP model and data mining* analyzes the data using statistical parameters about the machinery performance logs and sensor fault detection data to obtain information about the past and present. On the other hand, the use of naive Bayes algorithm at the *learning model* component allows tool condition monitoring for fault diagnosis at batch views component.

### 5.2. Real-Time Equipment Monitoring

The detection of anomalies in the process analysis in real-time for iCPS has allowed a new way of optimizing industrial systems supporting analysts and operators to solve possible problems [8].

Industrial data processes used to be streams obtained in real-time and, thus, fault detection methods must be able to handle the data in the temporal aspect, since it is only available at the window time of the acquisition. Table 4 presents different time assumptions for industrial temporal data for fault detection. Data streaming carries a set of values extracted from a data provider over time, such as sensor data collected from different aspects across manufacturing, including facilities equipment, process equipment, and physical defects.

In the scenario of the composition of data-driven events, real-time data determines active preventive maintenance, repair, and overhaul (MRO). Real-time monitoring at an earlier stage should prevent loss to machinery and reduce damage. In [35], a model-based technique is proposed that uses machine learning to identify internal faults of induction machines in real-time. Detection of device faults could have only minor manifestations but result in lower efficiency of operation. In that case, condition monitoring for inferring warning is quite useful to reduce industrial machine internal faults. In [36], a real-time fault monitoring system for industries is proposed to prevent severe machine damage in due course of time, based on random forest learning methods.

To illustrate one of many possible applications of the proposed architecture for industrial Big Data analytics in scenarios of real-time equipment monitoring, a real case reported in [37] is discussed below.

In [37], an autonomous learning system method for real-time fault detection in industrial processes based on TEDA (Typicality and Eccentricity Data Analytics) is proposed. The main advantages of the TEDA approach are that it does not require a priori knowledge about the streaming data, being this of particular importance in real-world applications; as well TEDA is very fast online, allowing its utilization for fault detection in the industry. To validate the scenario for real-time fault monitoring using TEDA for industrial problems in [37], real-world data from industrial plants were used. The open dataset DAMADICS (Development and Application of Methods for Actuator Diagnosis in Industrial Control Systems) was used in conjunction with a laboratory pilot plant. Data from DAMADICS comes from an actuator from the water evaporation process, which consists of the following components: control valve, pneumatic servomotor, and positioner. Data plant comes from several sensors and actuators controlled by programmable logical controller (PLC) that automates the flow between two tanks, two pneumatic control valves, and a centrifugal pump.

**Figure 13:**
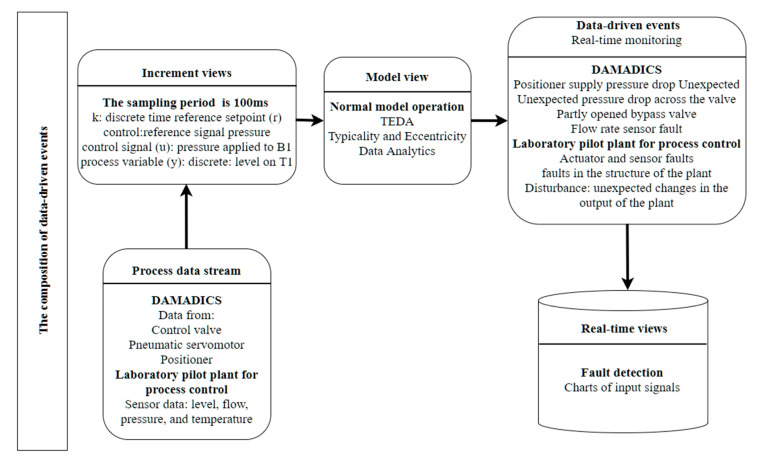
Instance of the real-time monitoring process for fault detection.

The process view of Figure 13 describes interactions between the architecture components for industrial analytics and the use case for real-time monitoring equipment activities that realize managing streaming data. Figure 13 shows an instance of the real-time monitoring process for fault detection. Data are processed in real-time from DAMADICS and the laboratory pilot plant for process control at the architecture component of the *process data stream*. At the architecture component of *increment views*, the data provider at time processing uses a temporary window to perform operations on the data. Data of the pilot plant are collected in a sampling period of 100 ms. That operation is intended to clean up the data, reduce the data frequency, and extract variables. Each data stream has monitored variables shown at the component of *increment views*. When tasks are completed, data variables are selected to be sent to the subprocess normal model operations for evaluation. In the component *real-time monitoring* of the *data-driven* *events* normal model operations are used to apply a multivariate learning model for underlying virtual dimensions that capture the “good” and in-control process data.

TEDA algorithm does not need any previous knowledge of data processes to learn to detect anomalies. Unsupervised learning methods do not need labeled data and detect rare events or outliers as data very distinct from the majority data based on identifying new types of anomalies as anomaly behavior analysis [34]. The detection and prediction processes use the prediction models generated during the information extraction process to identify patterns or predict states in real-time. The architecture component of a *data-driven event* monitors in real-time the submitted data and returns a prediction for an event in DAMADICS or the laboratory pilot plant for process control. This subprocess uses the model generated by the information extraction process to evaluate the data in real-time. The architecture component of *real-time views* displays charts of input signals for fault detection.

### 5.3. Forecasting Unusual Process Conditions

The scenario for forecasting unusual process conditions combines optimization data services and data-driven requirements for an adaptive health assessment. The efficiency improvement of asset-based, through real-time monitoring combined with the prediction of future behavior of machinery and performance log, allows health status monitoring of process conditions.

The fundamental premise is to develop a predictive model based on the sensor data log and apply the model to measure in real-time incoming stream data from sensors. The scenario challenge for the architecture design is twofold. First, to develop a learning model based on sensor Big Data. The second is to apply the analytics model to the stream measures of sensors. The Big Data challenge is to face intensive real-time data loads while a machine learning model is computed. The detection of anomalies, in this case, requires both the analysis of massive amounts of historical data and rapid processing based on intermediate results (anomaly detection model) [36].

An adaptive health assessment could be used to diagnose system reliability and forecast the machine condition, or a system based on health monitoring information. In [38], a generic methodology based on machine learning methods to strongly correlate faults detected in historical data of process log with the upcoming data stream, according to a prediction scope is presented. Data comes from the aluminum domain and represents the flow of the different phases and machine data comes from the process aluminum electrolysis and represents the flow of the different phases and machines to prepare the paste and form the anode (carbon blocks using for the aluminum reduction process). There are two data categories: the input signals are analyzed for the forecasting horizon, then the input data are correlated with the data of the internal system for fault diagnosis. The general approach consists of two parts: processing and applying the model to the ongoing process to detect outliers in underlying components and virtual dimensions.

**Figure 14:**
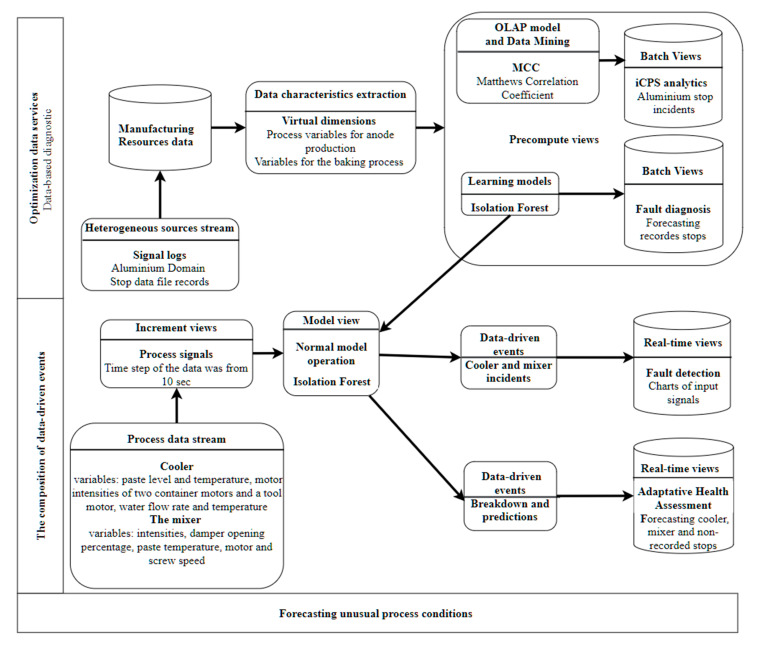
Instance for forecasting unusual process conditions for an adaptive health assessment.

Figure 14 shows an instance for forecasting an unusual process. In the part of data-based diagnostics, the architecture component of *manufacturing resources* stores data records from stop data files. The architecture component for the *data characteristics extraction* includes process variables for anode production and variables for the baking process. The use of Isolation Forest at the *learning model* component allows forecasting recorded stops for fault diagnosis at the batch view component. This model can also be applied to the *model view* component to monitor a breakdown event at process signals coming from the *process data stream* component. The source of streams includes variables from the cooler and mixer devices. That allows an adaptive health assessment at the architecture component of *real-time views* for the health forecasting of the cooler, mixer, and non-recorded stops.

Finally, it is also possible for the component *real-time process monitoring* to use normal model operations to apply a learning model that captures the “good” and in-control process data for fault detection.

## 6. Discussion, Research Limitations, and Further Work

This paper focuses on an architectural design that supports the integration of the IIoT environment, communications, and the cloud, in an analytics Big Data for data obtained from iCPS. The related works that are in the research scope are classified in (i) proposal of reference architectures in industrial contexts, and (ii) the architectural design of Big Data analytics for iCPS. Hence, a discussion is presented concerning research in each classification.

### 6.1. Reference Architectures in Industrial Contexts

The adoption of reference architectures accelerates the design of system solutions to common industrial process problems. However, proposals within the areas of Big Data are mainly focused on information systems or software in the field of business processes, being scarce those explicitly proposed for Industry 4.0. From literature related to reference architectures for industrial Big Data solutions, it has been observed that they do not provide systematic support for iCPS analytics towards generating possible architectural decisions alternatives according to industry business function. In [13], Big data-system design can use the architecture proposed in this paper as a reference architecture, and the components of each layer as design methods and technology catalog for Big Data analytics in industrial contexts. A limitation found in [7] shows that validation is required to account for prescriptive analytics in fault diagnosis scenarios of integrating iCPS with Big Data analytics. It has been presented the functional requirements and quality attributes required by stakeholders, to allow making analytics architectural design decisions for the detection of failure events in industrial plants. Compared with the 1-3-5 model in [14], the reference architecture proposed in this paper can be used as a guide to identify alternative architectural solutions that consider new data modeling analytics techniques which include diagnosis modeling, predictive modeling, and prescriptive modeling.

### 6.2. Big Data Analytics Architectures Design Applied in iCPS Contexts

The main reason for the complexity of developing Big Data analytics for iCPS is the difficulty in the integration of technologies in a consistent ecosystem in an industrial environment. Additionally, prescriptive analytics is inherently complex due to the need to align experience with model design, process optimization, and manufacturing forecasting. In the main literature review related to this topic, it has been observed that prescriptive analytics is not presented in them. For example, in [15], the model-driven proposal generates architectures for industrial data integration. In [10,16], a CBS adoption conceptual framework to support CPS manufacturing systems architecture for Industry 4.0 is defined. They focus particularly on guiding principles for CPS implementation in the industry through a methodology. Unlike those proposals, industrial Big Data attributes and data management drivers that specify architecturally significant requirements that guide the design of iCPS analytics architecture have been introduced in this paper.

In [17], the authors introduce an Industry 4.0 architecture like a general overview of components that include analytics. The main difference between the approach in this paper and the previous study, lies in narrowing the focus on analytics systems as the unit of the data model and design solution architectures to integrate them with data analytics platforms.

In the same way, the authors of [18] are trying to integrate the IIoT platform with Big Data analytics. A key feature is to enable IIoT for Big Data. While this approach acknowledges limitations in IIoT platforms enabling Big Data scenarios, and it keeps descriptions of process analytics, it does not represent the data management scenarios from manufacturing information systems. Nor do they address on-line analytical processing that allows structured analytics to support different perspectives in decision making. In [19], Big Data for prescriptive analytics is expected to be able to be used in manufacturing information systems (MIS) for advanced data analysis. Nevertheless, such work does not guide in an architectural framework for decision making in Big Data architecture design. The data management architecture proposed in this paper presents strong indications of being able to handle a wide range of industrial scenarios for prescriptive analytics, including fault diagnosis, real-time monitoring, and forecasting unusual process conditions, as it was discussed in the case study (see Section 5).

### 6.3. Research Limitations

As it has already been seen, manufacturing operations are affected by data management in several ways, however, industrial Big Data analytical projects used to suffer from long project execution time, making projects economically unattractive. It is believed that the proposed architectural based approach can significantly reduce the time spent developing Big Data analytics solutions for iCPS contexts. Although it is recognized that the fact of not presenting a real implementation of the proposed architecture could be considered a limitation of this work, it has been shown how it can be implemented with current open-source technologies. Moreover, the feasibility of the proposal to support the main functional and quality requirements was validated by use of the scenario technique, which is, perhaps, the most used approach to evaluate and validate software architectural proposals [28,30,31], since they help to know if the proposal could actually support the needed requirements before a costly real implementation, as it has been shown.

### 6.4. Further Work

The following significant research aspects in the architectural design for Big Data analytics in iCPS were found in the literature review and not addressed in this work, so they should be considered for future research:

- Decentralized control in iCPS
  The approach presented in this paper is under the scope of the centralized control paradigm that integrates iCPS, artificial intelligence, and Big Data analytics, and agent-based systems that will enable autonomous control of subsystems. An emerging trend is a decentralized control that uses adaptive models that support rapid model alteration that no manual upgrades are applicable. Such a new problem approach arises as the processes change over time in complex systems like the material flow of product parts and associated logistic operations. The question that arises is, how can Big Data analytics be integrated into the decentralized control approach in iCPS?
- iCPS analytics privacy concerns.
  An underlying concern in data security is industrial data privacy related to handling sensitive data without taking security measures, such as consideration of hidden Big Data analytics processing. An external server has become a potential security bridge to access confidential information from it. Thus, privacy concerns raised in Big Data analytics to concede data control to the cloud decrease confidentiality as data are probably stored, processed, and analyzed in several cloud centers leading security concerns into distinct locations of data. Then, the question that arises is, how does the Big Data analytics architecture handle sensitive data in iCPS contexts?

## 7. Conclusions

In this paper, a Big Data analytics architecture for iCPS was proposed. An ADD approach was adopted to gather industrial Big Data attributes (functional requirements) and industrial Big Data management drivers (data quality attributes) from planning, production, and maintenance, repair, and overhaul (different stakeholders) for the specification of the architecture. With those elements, the modeling of industrial Big Data design architecture for analytics was adopted, for predictive functions, inference of the key performance indicators, and data-driven events for real-time analysis.

From the functional point of view, the architecture is structured into the infrastructure layer, monitoring layer, and presentation layer. Additionally, component deployment was provided, covering all functional requirements using technologies that are accessible under an open-source license.

A case study for fault diagnosis conditions in industrial plants was discussed and used as a validation technique for the proposed architecture. It was performed through failure event detections to show the benefits of fault diagnosis, in-stream analytics, and the need to combine both for forecasting unusual process conditions, showing the feasibility of the usefulness of the proposed architecture. Finally, the contributions and strengths of the proposal compared to related works reported in the literature were discussed, as well as its limitations and future directions.

## Figures and Tables

**Table 1:** List of acronyms and their descriptions.

Acronym	Description	Acronym	Description
ADD	Attribute driven design	KPI	Key performance indicator
ALMA	Architecture level modifiability analysis	MES	Manufacturing execution system
ATAM	Architecture trade-off analysis method	MIS	Manufacturing information systems
CAD	Computer-aided design	MQTT	Message queue telemetry transport
CAE	Computer-aided engineering	MRO	Maintenance, repair and overhaul
CAM	Computer-aided manufacturing	OLAP	On-line analytical processing
CAS	Computer-aided systems	PaaS	Platform as a service
CBAM	Cost benefit analysis method	PDM	Precedence diagram method
CRM	Customer relationship management	PLC	Programmable logic controller
ERP	Enterprise resource planning	RFID	Radio frequency identification
ETL	Extract-transform-load	SAAM	Software architecture analysis method
HDFS	Hadoop distributed file system	SCADA	Supervisory control and data acquisition
iCPS	Industrial cyber-physical system	SCM	Supply chain management
IIoT	Industrial Internet of Things		

**Table 2:** Quality attributes for data management for industrial Big Data.

Quality Attributes		Description
Data source integration from iCPS	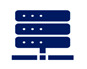	The IIoT in a smart factory generates large volumes of data and considering that data comes from heterogeneous sources such as programmable logic controller (PLCs), supervisory control and data acquisition (SCADA), enterprise resource planning (ERP), for the combination, integration, and later storage in large-scale Big Data, it requires an extract, transform, and load (ETL) system.
Data processing scalable and elastic	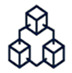	To ensure Big Data processing with very low latency in real-time coming from IoT technologies (smart sensors, radio frequency identification (RFID)), the hybrid cloud architecture must be scalable and elastic.
The composition of data-driven events	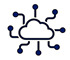	To provide a prescriptive analytics estimation from real-time data from IIoT technologies (smart sensors, RFID) provided from the expected manufacturing parameters in a reliable response time.
Optimization data services	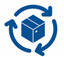	To provide a predictive analytics model from IIoT technologies (smart sensors, RFID) in hybrid clouds processing of Big Data for iCPS in a re-liable response time.
Embedded analytics	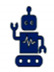	To provide specific algorithms of data analytics adapted to embedded hardware that produces insights close to the process/specific machine based on own generated data and data-at-rest sources in a reliable response time.
Analytics-based decision support	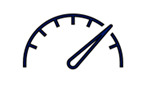	For business decision making through advanced prescriptive analytics and parametric analysis of business key performance indicators (KPIs) and estimate error/risk or predictions of these KPIs, it requires the integration of manufacturing data coming from IIoT technologies and manufacturing information systems (MIS).

**Table 3:** Requirements of fault diagnosis scenarios.

Requirement	Industrial Big Data Attribute	Scenario
Provide iCPS analytics with the comparison ability, where machinery performance logs can be compared with and rated among machines. Provide fault diagnosis of the machinery based on information about machine performance logs similarities.	Optimization data services	Fault diagnosis
Shift, drift, outliers in underlying components. The virtual sensor is working, and senses shift, drift, outliers. Root causes among parameters. Which specific parameters contribute most to the problem?	The composition of data-driven events	Real-time monitoring

**Table 4:** Time assumptions for industrial data.

Industrial Data	Data Source	Time Assumption
Facilities equipment	Sensor and environment data	Stream data
Process equipment	Sensor fault detection data	Stream data
Process results	Process history and measurements	Time series
Physical defects	Defect images and characteristics	Stream data
Product	Product test characteristics	Time series

## Data Availability

Not applicable.
